# Gastric leishmaniasis in the setting of HIV/AIDS infection at Community Hospital in Southeastern United States

**DOI:** 10.1099/acmi.0.000045

**Published:** 2019-07-08

**Authors:** Dontre' M. Douse, Randi S. Goldstein, David J. Montgomery, Michael Sinnott

**Affiliations:** ^1^ Mercer University School of Medicine, GA, USA; ^2^ Department of Pathology, Memorial Health University Medical Center, Savannah, GA, USA

**Keywords:** Leishmania, leishmaniasis, gastric leishmaniasis, HIV/*Leishmania* co-infection

## Abstract

**Introduction:**

Visceral leishmaniasis, caused by the *Leishmania donovani* complex, is responsible for over 20 000 deaths per year. This disease often affects the immunocompromised with an increased prevalence in those with human immunodeficiency virus (HIV). The immunocompromised are not only more susceptible to infection, but disseminated disease including gastric leishmaniasis. This is a case of gastric leishmaniasis occurring in a non-endemic region in a patient with comorbid HIV.

**Case presentation:**

The patient is a 39 year old originally from Central America currently living in Southeast Georgia. His history is significant for HIV, alcohol abuse, tobacco dependency and bone marrow biopsy-proven leishmaniasis. He denied any recent travel. At initial presentation, he had abdominal pain, nausea/vomiting, chills and dysphagia along with leukopenia and thrombocytopenia. Treatment with amphotericin B was initiated for his leishmaniasis as well as highly active antiretroviral therapy (HAART). The patient was discharged home on a 3 month course of amphotericin B with continued HAART therapy. Following resolution of his acute symptoms, six months later, the patient developed acute abdominal pain with nausea prompting presentation to the emergency department. Leishmaniasis was found again following bone marrow biopsy and the patient restarted amphotericin B and HAART. Several years later the patient presented again with similar symptoms, this time with accompanying rectal bleeding. The patient received an esophagogastroduodenoscopy and on gastric mucosal biopsy was found to have gastric leishmaniasis.

**Conclusion:**

This manuscript highlights the key features of this case, including recognizing leishmaniasis clinically, proving diagnosis through definitive testing and understanding the connection between leishmaniasis and HIV.

## Introduction

Visceral leishmaniasis, also known as kala-azar, is a disease caused by the *Leishmania donovani* complex. According to the World Health Organization, visceral leishmaniasis is responsible for 20 000 to 40 000 deaths per year [1]. It is a blood-borne infection most often transmitted by its vector, the phlebotomine sandfly or needle sharing [2]. As with other communicable illnesses, geography plays a pivotal role in this disease process as more than 90 % of new cases in 2015 occurred in seven countries: Brazil, Ethiopia, India, Kenya, Somalia, South Sudan and Sudan [1, 3]. With this thought in mind, it is not surprising that the taxonomy of these parasites is decided based upon geography. For example, *L. donovani* is predominantly found in South Asia and East Africa, while *L* *e* *i* *shmania infantum* is prevalent in the Mediterranean basin, western Asia and parts of the Middle east [4]. According to Lindoso, human immunodeficiency virus (HIV) is responsible for increasing the spread of visceral leishmaniasis [3]. HIV causes individuals to be in an immunocompromised state increasing susceptibility to not only develop leishmaniasis, but disseminated leishmaniasis. In fact, they are at risk for an uncommon presentation, gastric leishmaniasis [5, 6]. Presented below is a case of gastric leishmaniasis occurring in a non-endemic region in a patient with comorbid HIV.

## Case report

The patient is a 39-year-old gentleman originally from Central America who currently lives in Southeast Georgia. His past medical history is significant for HIV/acquired immunodeficiency syndrome (AIDS), alcohol abuse, tobacco dependency and bone marrow biopsy-proven leishmaniasis who was transferred from an outside hospital. He denied any recent travel. At first presentation, the patient had diffuse moderate abdominal pain, nausea, vomiting, chills and dysphagia. On physical exam, the patient was tachycardic to a rate of 140 beats per minute and the patient was found to have mild abdominal tenderness, but the physical exam was otherwise non-contributory. The patient was leukopenic as well as thrombocytopenic. For treatment, the patient was placed on amphotericin B for his leishmaniasis as well as highly active antiretroviral therapy (HAART) for his HIV/AIDS infection. The patient was then discharged home on a 3 month course of amphotericin B in addition to continued HAART therapy with outpatient follow-up.

**Fig. 1.**
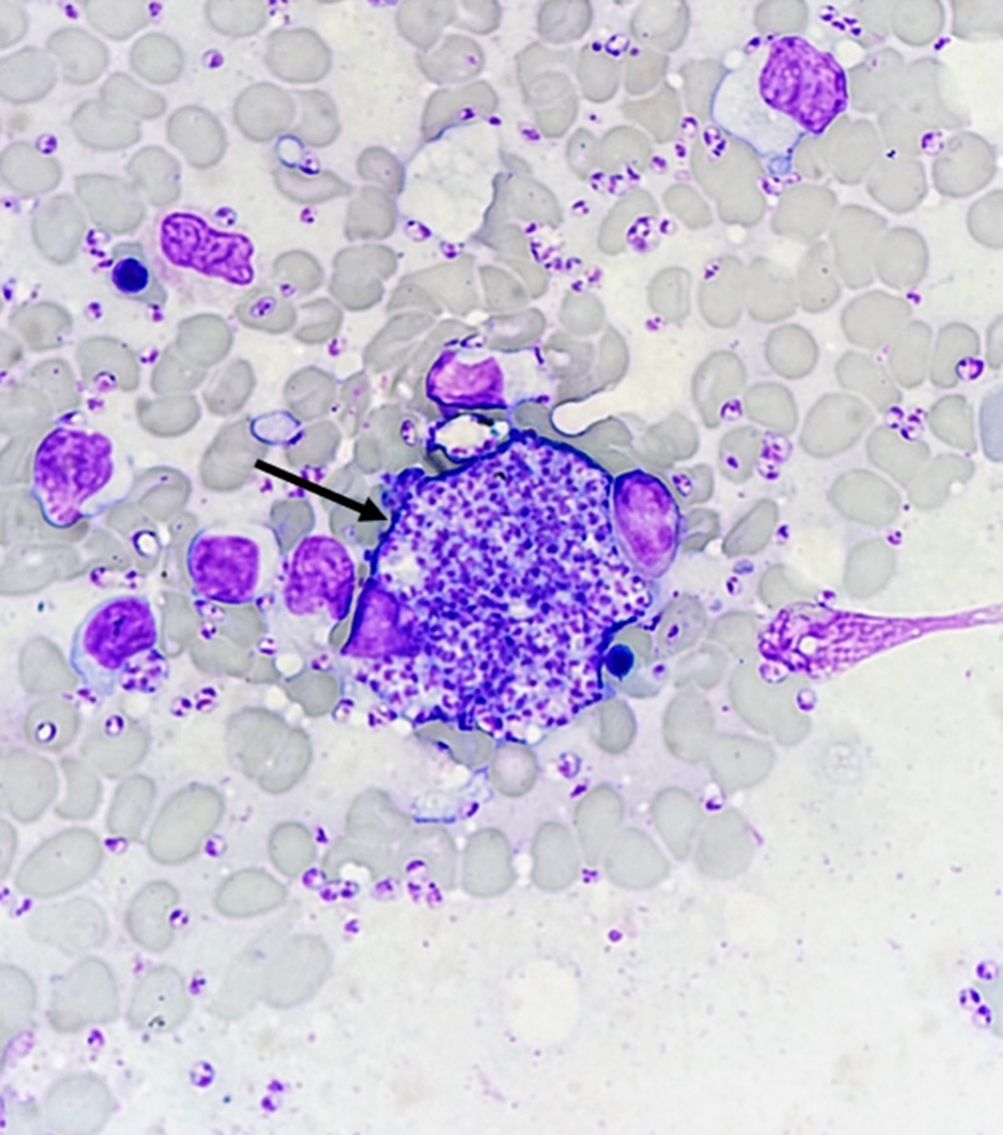
Bone marrow smear preparation showing a macrophage filled with *Leishmania amastigotes* (central black arrow).

**Fig. 2.**
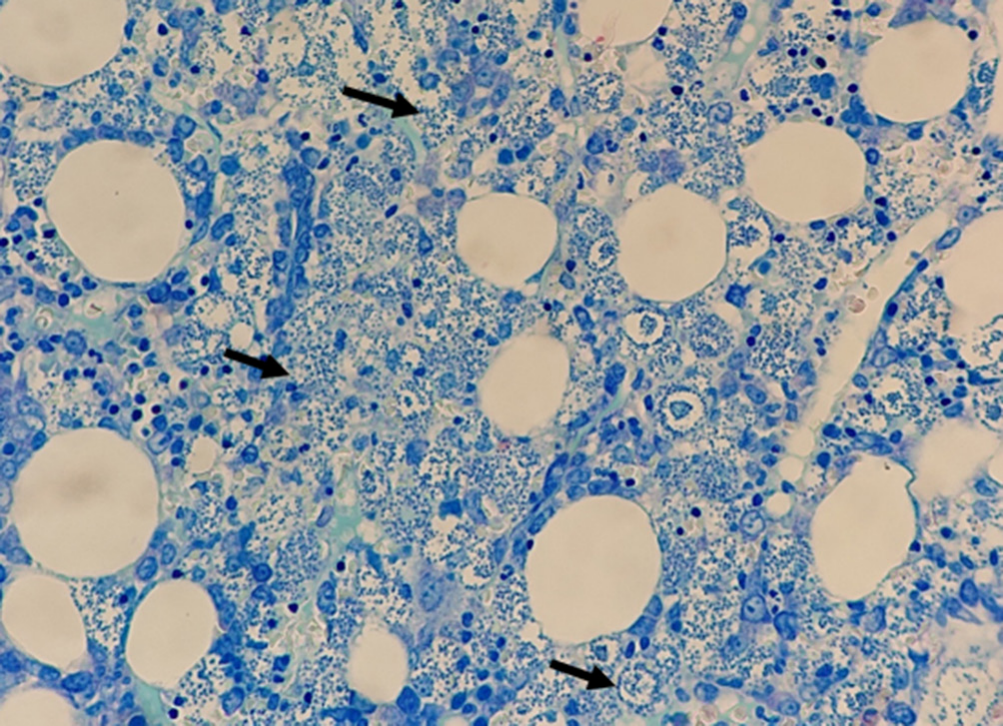
Photomicrograph of the bone marrow using Giemsa special stain, highlighting the macrophage-filled *L. amastigotes* (black arrows).

**Fig. 3.**
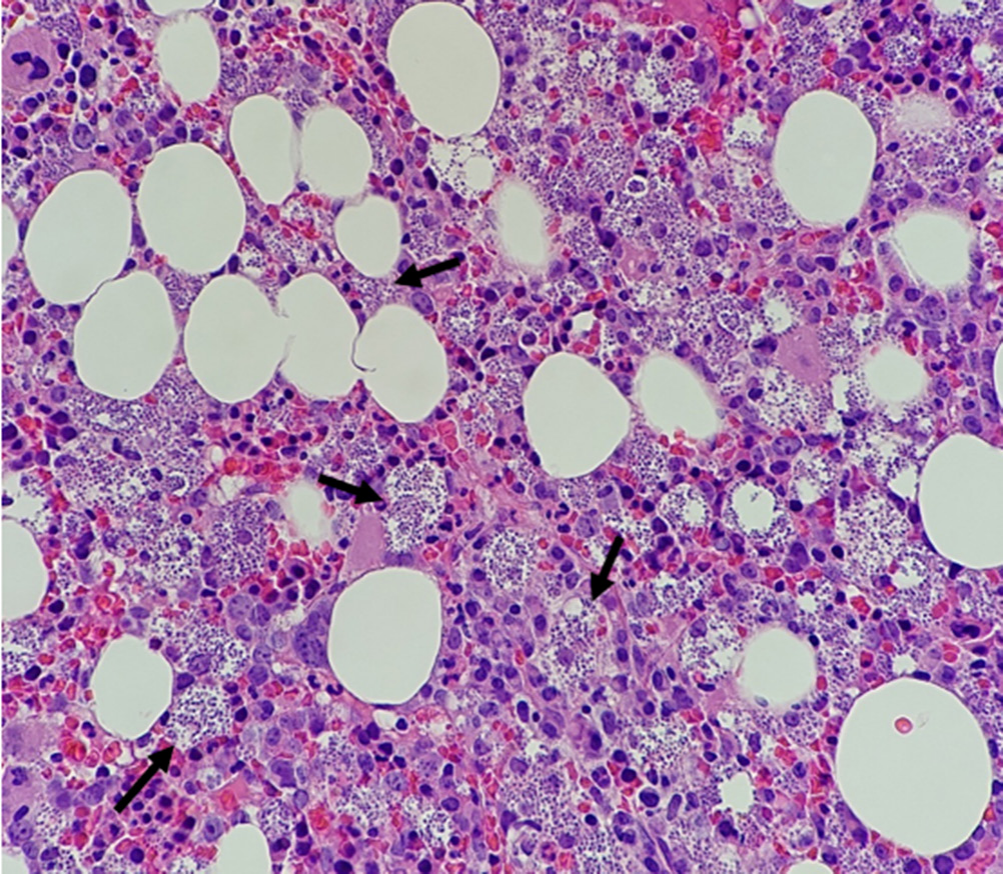
Photomicrograph of bone marrow showing numerous macrophages containing *L. amastigotes* (black arrows).

Over the course of the next 6 months, the patient initially recovered from his acute illness, but later developed acute abdominal pain and tenderness along with nausea, which prompted presentation to the emergency department. On exam, the patient was found to have a fine nodular skin rash across his chest, abdomen and parts of his back consistent with cutaneous leishmaniasis. Additionally, he was found to have continued thrombocytopenia. A bone marrow biopsy was done to differentiate whether the new onset symptoms were secondary to his HIV or related to a relapse of the his leishmaniasis. The bone marrow results are shown in Figs 1–3 *,* which demonstrates various stains showing the presence of macrophages filled with Leishmaniasis amastigotes. The patient was again initiated on intravenous amphotericin B for 6 weeks inpatient and discharged with the plan to get an amphotericin B infusion every 3 weeks on an outpatient basis. The patient was steadily improving on his inpatient treatment and was cleared for discharge home, the patient, however, was lost to follow-up.

**Fig. 4.**
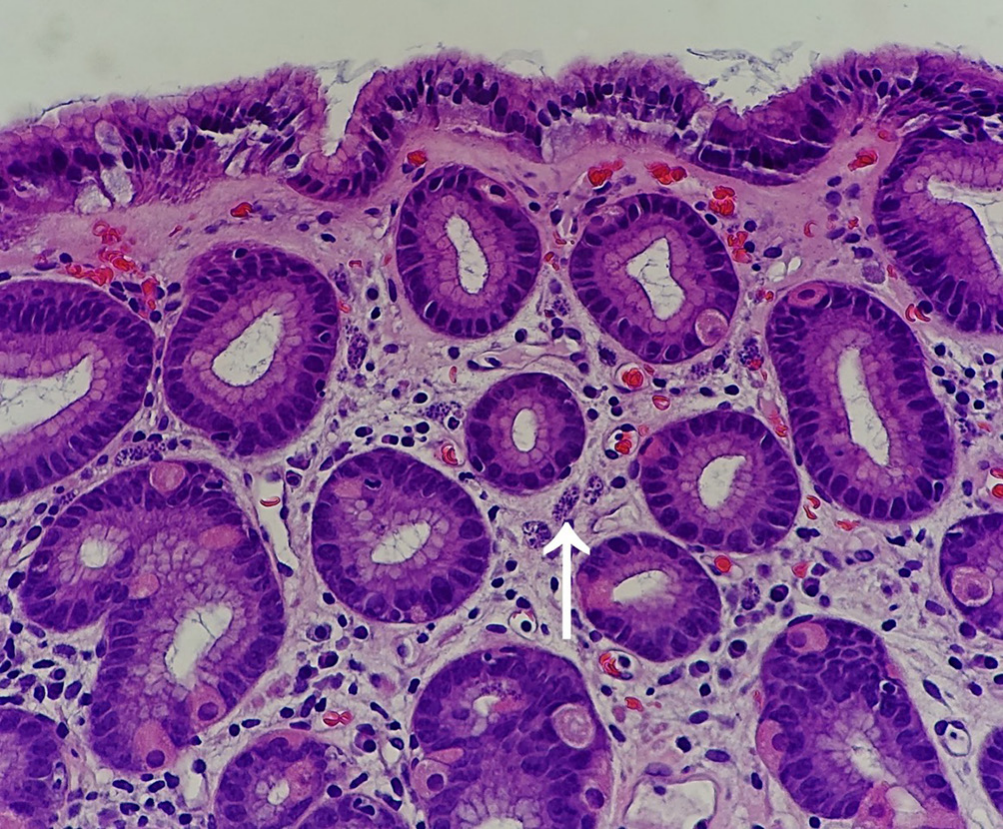
Photomicrograph of the gastric mucosa showing scattered macrophages (white arrow) containing *L. amastigote*s.

Several years later the patient returned to the emergency department with the most recent flare of his Leishmaniasis disease. The patient presented with rectal bleeding accompanied by continued abdominal pain and discomfort. This hospital course, the gastroenterology team was consulted. An esophagogastroduodenoscopy was performed, and a biopsy was taken of his gastric mucosa. The biopsy showed scattered macrophages filled with amastigotes throughout the patient’s gastric mucosa (*see* Fig. 4). These findings confirmed the suspected case of gastric leishmaniasis. The patient was subsequently treated with amphotericin B to treat his disseminated gastric leishmaniasis and encouraged to stay consistent with his HAART for his HIV. The patient responded well, his gastrointestinal bleed resolved, and the patient clinically had no complaints or concerns. The patient was encouraged to follow-up in the outpatient setting with his gastroenterologist for his gastric leishmaniasis and primary care physician for his HIV/AIDS disease.

## Discussion

Based upon the literature, the case presentation is fairly typical for gastric leishmaniasis with an HIV co-infection. According to Kamboj *et al*. the typical presentation for visceral leishmaniasis is one affecting the spleen, bone marrow and liver, however with a co-infection with HIV the patients may have an atypical presentation of predominantly gastrointestinal involvement [2]. Described in a case report from 2010, Warich-Eitel *et al*. describe a similar set of symptoms of fatigue, thrombocytopenia, intermittent epistaxis and the presence of upper gastrointestinal bleeding [5]. This vague symptomatology is one that can fit a myriad of various etiologies leading to a difficulty in diagnosis. This presentation of gastric leishmaniasis co-infected with HIV has been previously identified in southern Europe with presumed prevalence across areas with larger HIV populations [5]. In 1994, 3–7 % of patients infected with HIV-1 in southern Europe had a co-infection of gastric leishmaniasis [7]. This patient’s presentation is typical for the presentation of gastric leishmaniasis but his lack of a history of travel to endemic areas presents a curious conundrum. Since the patient is originally from Guatemala and he only speaks Spanish, the possibility of an incomplete travel history due to the language barrier is certainly evident. With growing trends of rapid and distant travel the importance of keeping previously region-specific infections in mind becomes increasingly imperative to the prevention of disease sequelae. According to the last published data from the WHO in 2010, zero cases of cutaneous leishmaniasis were reported in the United States that year [1]. This fact alone is evidence as to why leishmaniasis is rarely on the differential diagnosis for patients especially ones presenting with abdominal pain. Justly, this is often not considered due to its infrequency; however, if left untreated this infection is fatal and emphasizes the importance of utilizing the proper diagnostic studies when evaluating a patient presenting with a gastrointestinal bleed. As with other parasitic infections, the diagnosis of leishmaniasis is dependent upon direct biopsy of the parasitic lesion. Warich-Eitel *et al*. describe a 43-year-old HIV-infected patient diagnosed with gastric leishmaniasis via histopathology as an accidental finding. Leishmania gastritis is a rare disease that can occur without any signs or symptoms and without any endoscopic abnormalities. Thus, microscopic detection of the pathogen inside macrophages of gastric mucosa is required for accurate diagnosis [5].

Gastric leishmaniasis has been well attributed to patients in an immunocompromised state and this association is thought to be the mechanism behind the co-infection often seen with HIV. This etiology is further reinforced by the increasing prevalence of gastric leishmaniasis seen in other immunocompromised patients such as those who have previously had an organ transplant or are on an immunosuppressant for another reason [8]. According to Olivier *et al*. leishmaniasis may not only be an opportunistic infection secondary to an immunocompromised state but rather seems to propagate the progression of HIV-1 to AIDS. Leishmania is an intracellular protozoan parasite. Both HIV-1 and leishmania invade and multiply inside cells of the myeloid or lymphoid lineage. *L. donovani* can up regulate HIV-1 replication *in vitro* and in co-infected individuals, providing evidence to support the idea that leishmania likely acts as a strong co-factor in the pathogenesis of HIV-1 infection. Olivier *et al*. also describe an observation that the surface lipophosphoglycan (LPG) on *L. donovani* could induce HIV-1 replication in several monocytoid cell lines chronically infected with HIV-1. LPG also mediates protection of the parasite inside the macrophage phagolysosome and induces inhibition of macrophage function, including generation of oxygen radicals. HIV infection induces immunosuppression, which may permit re-activation of latent leishmanial infections and may promote the development of visceral leishmaniasis. Since both the leishmania parasite and HIV invade and replicate inside the same cells, it is possible that the interactions between these two pathogens could exacerbate both infections, having a synergistic response [**7**]. Another key feature of this case is the nature of this patient’s recurrent episodes of leishmaniasis. This patient over the course of several years dealt with either incompletely treated or treatment refractory leishmaniasis. This phenomenon is a relatively often reported occurrence in this patient population often attributed to the severely immunocompromised state of patients dealing with a chronic HIV infection. Thus, in patients with a significant history of leishmaniasis, a high degree of suspicion must remain as to gastric leishmaniasis being a possible etiology to an atypical progression of HIV/AIDS.

Diagnosis of leishmaniasis is often secondary to a high suspicion of exposure to the infective vector. With the patient in this case report, the initial diagnosis of leishmaniasis had been made prior to initial presentation. This prior history of leishmaniasis is what gave the high degree of suspicion for gastric leishmaniasis. This is often not the case and the diagnostician must remain vigilant for a parasitic etiology in cases with such vague symptomatology. The diagnosis of gastric leishmaniasis as with the other forms is based upon direct visualization of the parasite using a Giemsa stain. Similarly, to other parasitic organisms, *L. donovani* is collected through biopsy or scraping of a cutaneous lesion. Although an unlikely cause of an upper gastrointestinal bleed, parasitic infection is a potentially treatable source that can provide significant relief for the patient.

## Conclusion

This case report describes an uncommon presentation of leishmaniasis in a non-endemic area that was found to be refractory to treatment. Although gastric leishmaniasis has been associated with HIV co-infection, the occurrence of this illness in a non-endemic area is quite uncommon. As described above, these two illnesses have a synergistic effect when co-infected, often expediting the severity of one another leading to an increased mortality unless identified early. The refractory nature of this illness when in the setting of an HIV co-infection emphasizes the importance of close follow-up of the patients due to the lethal nature if left untreated. Since this infection is quite unlikely in the United States, physicians must be vigilant in terms of keeping the possibility of a potential zebra on differential diagnosis especially in the setting of an HIV-positive patient.

In conclusion, this manuscript highlights the key features of this case, including (1) leishmania diagnosis in a non-endemic area, (2) gastric leishmania in patients with HIV, (3) need for biopsy for diagnosis and (4) to urge providers to keep leishmania on the differential in the setting of an immunocompromised HIV patient with abdominal symptoms.
